# Impact of age at diagnosis of head and neck cancer on incidence of metachronous cancer

**DOI:** 10.1186/s12885-018-5231-7

**Published:** 2019-01-03

**Authors:** Taro Iwatsubo, Ryu Ishihara, Toshitaka Morishima, Akira Maekawa, Kentaro Nakagawa, Masamichi Arao, Masayasu Ohmori, Hiroyoshi Iwagami, Kenshi Matsuno, Shuntaro Inoue, Hiroko Nakahira, Noriko Matsuura, Satoki Shichijo, Takashi Kanesaka, Sachiko Yamamoto, Yoji Takeuchi, Koji Higashino, Noriya Uedo, Isao Miyashiro, Kazuhide Higuchi, Takashi Fujii

**Affiliations:** 1 Department of Gastrointestinal Oncology, Osaka International Cancer Institute, 3-1-69 Otemae, Chuo-ku, Osaka, 541-8567 Japan; 2 Second Department of Internal Medicine, Osaka Medical College, Osaka, Japan; 3 Department of Cancer Strategy, Osaka International Cancer Institute, Osaka, Japan; 4 Department of Head and Neck Surgery, Osaka International Cancer Institute, Osaka, Japan

**Keywords:** Cumulative incidence, Esophageal cancer, Head and neck cancer, Metachronous cancer, Young onset

## Abstract

**Background:**

Metachronous cancer in patients with head and neck cancer (HNC) is common and is associated with a poor prognosis. We aimed to evaluate the incidence of metachronous cancer at different sites according to age at diagnosis of index HNC.

**Methods:**

We collected data on 2011 patients with oral cancer, oropharynx cancer, hypopharyngeal cancer, and laryngeal cancer as index cancers using the Osaka International Cancer Institute Cancer Registry database between 2005 and 2016. Among these, we analyzed 1953 patients after excluding 5 patients who were not followed-up and 53 patients with simultaneous multiple index cancers. We evaluated the cumulative incidence of metachronous cancer in the esophagus, lung, and other sites according to age at diagnosis of the index HNC using the Kaplan–Meier method. Multivariate logistic regression analysis was performed to identify factors that influenced the incidence of metachronous cancers following HNC.

**Results:**

The cumulative incidence of metachronous esophageal cancer in young patients (< 65 years) was significantly higher than that in old patients (≥ 65 years) (12.1% vs 8.5% at 5 years, and 16.5% vs 11.2% at 10 years; *p* = 0.015). On the other hand, the cumulative incidence of the other cancers in young patients was significantly lower than that in old patients (7.8% vs 12.2% at 5 years, and 13.9% vs 15.3% at 10 years; *p* = 0.017). The cumulative incidence of lung cancer was not significance according to age at diagnosis of the index HNC. In the multivariate analysis, histological type (squamous cell carcinoma) and lesion location (hypopharynx and larynx) were independently associated with metachronous cancers. Moreover, age at diagnosis of the index HNC (< 65 years), histological type (squamous cell carcinoma) and lesion location (hypopharynx) were significant predictors of metachronous esophageal cancer incidence and lesion location (hypopharynx) was a significant predictor of metachronous lung cancer incidence.

**Conclusion:**

Risk stratification of metachronous cancers with age and other predictors may help to properly manage patients with HNC.

**Trial registration:**

The present study is a non-intervention trial.

## Background

Head and neck cancer accounts for more than 900,000 cases and 370,000 deaths worldwide annually [1]. In Japan alone, head and neck cancer accounts for more than 39,000 cases and approximately 10,000 deaths from the disease annually [2, 3]. The risk of second primary cancer (SPC) in patients with head and neck cancer (HNC) is higher compared to the age-matched general population [4, 5]. In previous reports, the incidence of SPC following HNC was 3–4% per year [6–8]. The overall survival rates of patients with SPC were lower than those for patients without SPC, especially those with SPC in the esophagus or lungs [6, 7, 9, 10]. Therefore, the diagnosis and treatment for metachronous esophageal and lung cancer following HNC is important.

When synchronous and metachronous squamous cell carcinoma develop within the upper aerodigestive tract (including the lips, mouth, tongue, throat, vocal cords, and part of the esophagus and trachea), the phenomenon is referred to as “field cancerization” [11]. Epidemiological studies have consistently demonstrated that strong alcohol consumption and heavy smoking are important risk factors for HNC and esophageal cancer [12–15]. Based on these findings, the World Health Organization (WHO) categorized alcohol consumption and smoking as a group I carcinogen for the oral cavity, pharynx, larynx, and esophagus [16].

Since the overall survival rate in patients with HNC differs according to SPC condition, we should recognize the high-risk population for SPC and focus on the development of SPC during the surveillance period after curative treatment for HNC.

In general, older people may be at a high risk of developing any kind of cancer. However, in clinical practice, SPCs in patients with HNC are frequently observed in young populations. Knowing the incidence of SPCs with respect to age may provide important information on the risk stratification of SPCs in HNC patients. The cancer registry integrates histology, treatment, drug, and accounting databases, as well as includes the dates of diagnosis and treatment. By combining multiple databases, the cancer registry can accurately identify HNC patients with or without SPCs. The aim of this study was to clarify the age-specific incidence of SPCs, especially esophageal cancer, in patients with HNC, based on hospital cancer registry data.

## Methods

### Study population

The present study is a retrospective cohort study at a single center. The study subjects were patients with HNC, including oral cancer, oropharynx cancer, hypopharyngeal cancer, and laryngeal cancer, prospectively recorded as an index cancer in the Osaka International Cancer Institute Cancer Registry between January 2005 and December 2016. This record includes information about patients’ sex, age at diagnosis, sequential order of cancer incidence, histological type of cancer, and cancer site categorized according to the third edition of the International Classification of Diseases for Oncology (ICD-O-3), such as mouth/pharynx (C00–14), esophagus (C15), larynx (C32), and lung (C33, C34) [17]. We excluded patients with simultaneous multiple cancers because we could not identify which cancer was an index cancer. In addition, we excluded patients whose observation period was only the day of diagnosis of the index cancer. The study protocol was approved by the Institutional Review Board at our center (No. 1803309441).

### Outcomes

The primary outcome was cumulative incidence of SPC in patients with HNC and age-specific cumulative incidence of cancer (at diagnose of the index cancer). Initially, oral cancer, oropharynx cancer, hypopharyngeal cancer, and laryngeal cancer were defined as index cancers. Moreover, considering the difference in the influence of carcinogens, SPCs were classified into three categories: (1) esophageal cancer; (2) lung cancer; and (3) other cancers. Esophageal cancer is strongly associated with exposure to carcinogens such as alcohol and tobacco smoke, lung cancer is associated with tobacco smoke and other cancers are less influenced by alcohol and tobacco smoke. Development of SPCs in each category was separately analyzed.

### Statistical analysis

Continuous variables are presented as the mean and standard deviation (SD) or median and range, as appropriate for the data type. A chi-squared or Fisher’s exact test was used for analyzing categorical variables. Probability values for statistical tests were two-tailed and *p* < 0.05 was considered significant. The incidence time curve in each group was generated using the Kaplan–Meier method. Multivariate logistic regression analysis was performed to identify factors that influenced the incidence of SPCs following HNC. Factors that could be extracted from the cancer registry database (sex, age, histological type of cancer, and cancer site categorized according to the ICD-O-3) were included in the analyses. All analyses were performed using the statistical program “R” version 3.3.3 (R Foundation, Vienna, Austria).

## Results

**Fig. 1:**
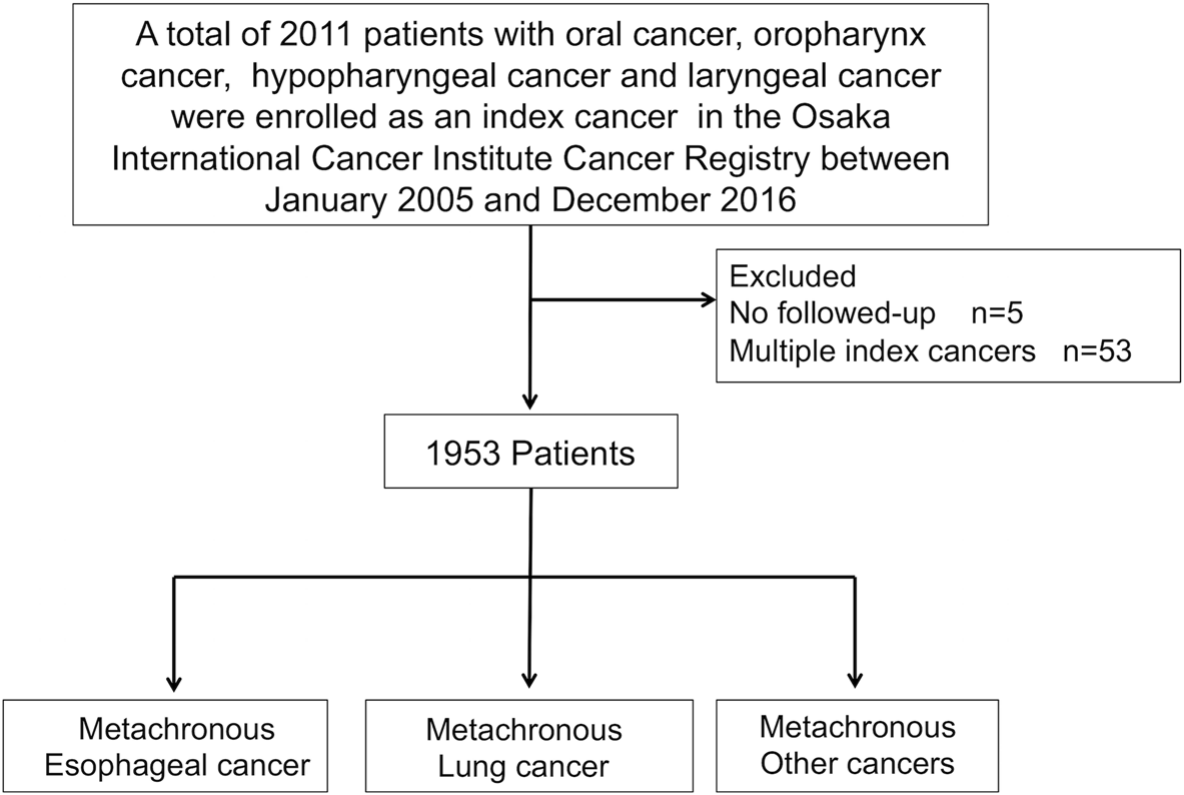
Flow diagram of enrollment in this study

**Fig. 2:**
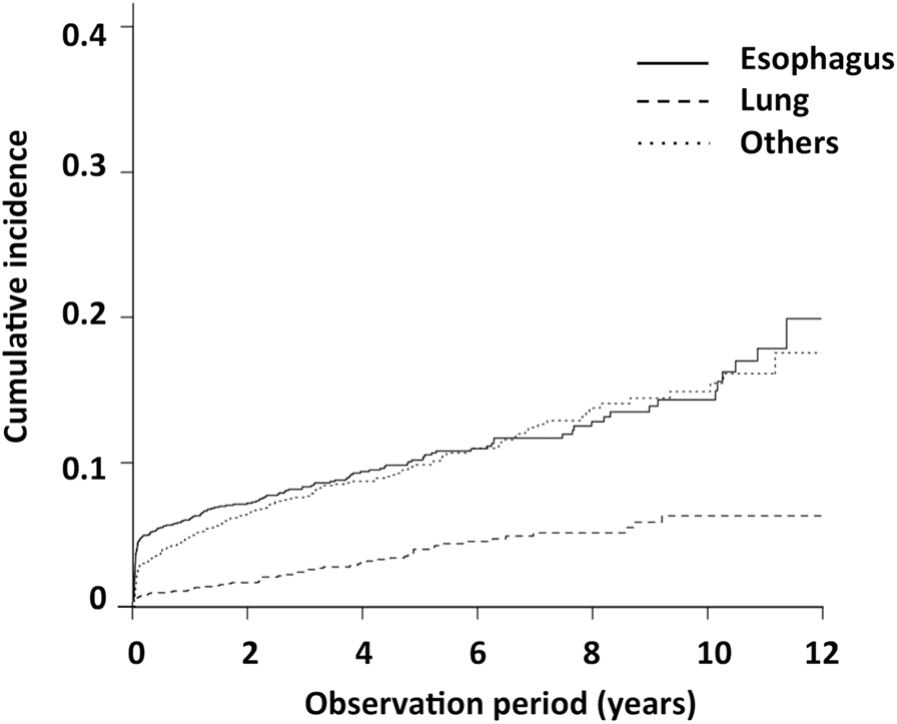
Cumulative incidence of second primary cancers after index head and neck cancers according to cancer site

We identified 2011 patients who developed oral cancer, oropharynx cancer, hypopharyngeal cancer, and laryngeal cancer as index cancers using our hospital cancer registry and included 1953 patients with new HNCs, without a past history of any other cancers in the analyses (Fig. 1). The background characteristics of patients at diagnosis of the index cancers are summarized in Table 1. The cumulative incidence of SPCs according to cancer site (esophagus, lungs, and others) is presented in Fig. 2 and is as follows: 10.4, 4.0, and 9.8% at 5 years; and 14.2, 6.3, and 14.8% at 10 years, respectively, after diagnosis of the index cancers. A total of 190 patients developed metachronous esophageal cancers after a median follow-up period of 41 months (range 0–154); 73 patients were older than the median age of 65 years and 117 patients were below the median age (Table 2).

**Table 1:** Patient characteristics

All cases, n	1953
Gender, male/female, n	1588/365
Age, median (range), year	65 (12–97)
≥ 65, n	983
< 65, n	970
Lesion location	
Oral cavity, n (%)	514 (26)
Oropharynx, n (%)	300 (15)
Hypopharynx, n (%)	541 (28)
Larynx, n (%)	598 (31)
Histological type	
Squamous cell carcinoma, n (%)	1863 (95.4)
Malignant lymphoma, n (%)	28 (1.4)
Basaloid squamous cell carcinoma, n (%)	9 (0.4)
Verrucous carcinoma, n (%)	8 (0.4)
Adenoid cystic carcinoma, n (%)	7 (0.4)
Malignant melanoma, n (%)	5 (0.3)
Neuroendocrine carcinoma, n (%)	4 (0.2)
Papillary squamous cell carcinoma, n (%)	3 (0.2)
Mucoepidermoid carcinoma, n (%)	3 (0.2)
Others, n (%)	14 (0.7)
Unknown, n (%)	9 (0.4)

**Table 2:** Incidence of metachronous cancer after head and neck cancer by age at diagnosis of index cancer

	Age ≥ 65 *n* = 983	Age < 65 *n* = 970	Total *n* = 1953
Esophageal cancer, n	73	117	190
Lung cancer, n	37	28	65
Other organs			
Gastric cancer, n	39	33	72
Colorectal cancer, n	17	18	35
Liver cancer, n	9	6	15
Prostatic cancer, n	7	4	11
Thyroid cancer, n	4	7	11
Renal and ureter cancer, n	8	2	10
Breast cancer, n	4	3	7
Pancreatic cancer, n	3	1	4
Uterine cancer, n	1	1	2
Others, n^a^	5	6	11
Total, n	97	81	178

^a^Others include 3 lymphomas (1 eye socket, 2 lymph nodes, and 1 unknown region), 2 myelomas, 1 ovarian cancer, 1 cancer of the maxillary sinus, 1 duodenal cancer, and 1 cancer of unknown primary origin

**Fig. 3:**
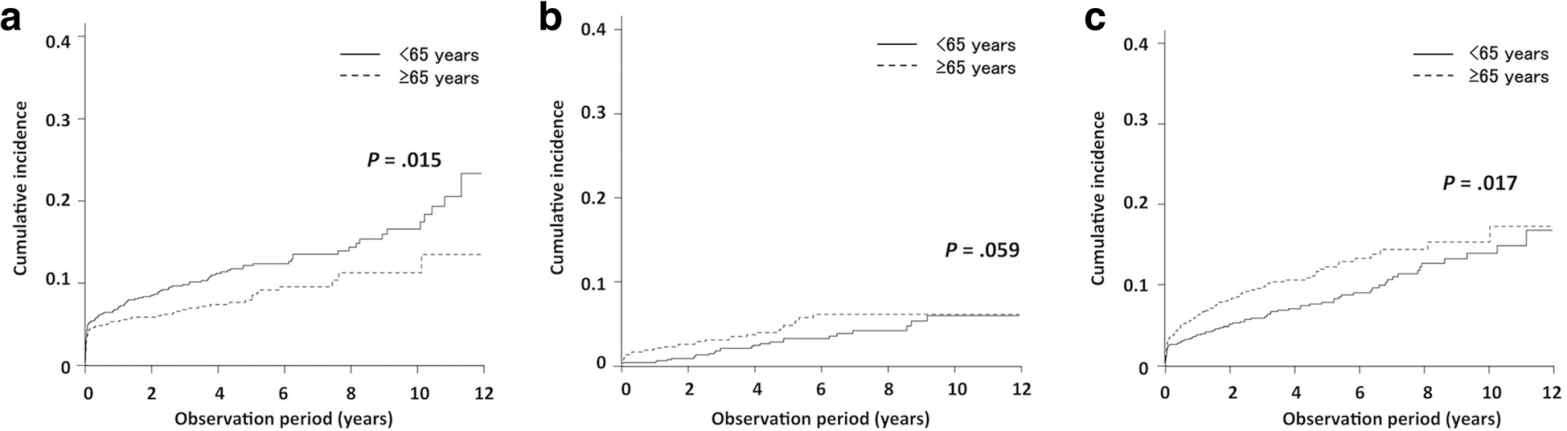
Cumulative incidence of metachronous (**a**) esophageal cancer, (**b**) lung cancer and (**c**) other organ cancers according to age at diagnosis of index head and neck cancers

The cumulative incidence of metachronous esophageal cancers according to age at diagnosis of the index cancers is presented in Fig. 3a. Consequently, the cumulative incidence in old versus young patients was 8.5% vs 12.1% at 5 years, and 11.2% vs 16.5% at 10 years, respectively (*p* = 0.015). The cumulative incidence of lung cancer in old versus young patients was 4.8% vs 3.3% at 5 years, and 6.1% vs 6.0% at 10 years, respectively (*p* = 0.059, Fig. 3b), while the cumulative incidence of the other cancers in old versus young patients was 12.2% vs 7.8% at 5 years, and 15.3% vs 13.9% at 10 years, respectively (*p* = 0.017, Fig. 3c). The multivariate analyses of variables predictive of SPC following HNCs are shown in Table 3. Histological type (squamous cell carcinoma) and lesion location (hypopharynx and larynx) were independently associated with all cancers. Moreover, age at diagnosis of the index HNC (< 65 years), histological type (squamous cell carcinoma) and lesion location (hypopharynx) were significant predictors of metachronous esophageal cancer incidence and lesion location (hypopharynx) was a significant predictor of metachronous lung cancer incidence.

**Table 3:** Multivariate predictors for incidence of metachronous cancers after head and neck cancer

		All cancers			Esophageal cancer			Lung cancer			Other cancers		
		OR	95%CI	*P* value	OR	95%CI	*P* value	OR	95%CI	*P* value	OR	95%CI	*P* value
Age	< 65 years	1.20	0.95–1.52	0.131	1.75	1.27–2.41	< 0.001	0.84	0.50–1.39	0.492	0.89	0.65–1.22	0.465
	≥65 years	1			1						1		
Sex	Male	1.32	0.94–1.87	0.129	1.20	0.69–1.74	0.697	2.02	0.78–5.22	0.149	1.65	0.99–2.72	0.051
	Female	1			1			1			1		
Histological type	Squamous cell carcinoma	3.63	1.30–10.1	0.014	7.50	1.02–55.2	0.048				2.17	0.67–7.05	0.196
	Others	1			1			Not applicable^a^			1		
Lesion location	Oral cavity	1			1			1			1		
	Oropharynx	1.08	0.68–1.72	0.731	1.40	0.72–2.71	0.325	0.33	0.07–1.56	0.162	0.88	0.50–1.57	0.669
	Hypopharynx	3.17	2.25–4.48	< 0.001	5.88	3.62–9.55	< 0.001	2.47	1.13–5.42	0.024	1.20	0.76–1.88	0.439
	Larynx	1.92	1.34–2.74	< 0.001	1.53	0.88–2.68	0.132	2.14	0.97–4.73	0.061	1.42	0.91–2.20	0.122

*OR* Odds ratio, *CI* confidence interval

^a^Histological type was excluded from the multivariate analysis for metachronous lung cancer which developed only in patients with head and neck squamous cell carcinoma

## Discussion

The cumulative incidence of most types of cancer generally increased with age at diagnosis of the index HNCs. However, the result for esophageal cancer was the opposite, i.e., young patients (< 65 years) were more likely to develop metachronous esophageal cancer than old patients (≥65 years). In the multivariate analysis, young-onset (< 65 years), squamous cell carcinoma and hypopharyngeal cancer were identified as predictors for developing metachronous esophageal cancer. To the best of our knowledge, this is the first report comparing the difference in the cumulative incidence of metachronous esophageal cancer, lung cancer, and other cancers by evaluating index HNCs between young and old patients using a hospital cancer registry. The cancer registry is a database of accurate information on the diagnosis of cancer by integrating histology, treatment, drug, and accounting databases. The registry aids in comprehensively examining a vast range of information on cancer across each organ in a large cohort.

In this study, incidence of SPC was associated with histological type (squamous cell carcinoma) and lesion location (hypopharynx and larynx), but not associated with age. The main risk factors of hypopharyngeal and laryngeal cancer are alcohol and cigarette smoke [13, 15, 16] . Excessive drinking and smoking in patients with hypopharyngeal and laryngeal cancer are also risk factors for cancer development in other organs and likely the cause of increased SPC risk in these patients. On the other hand, other factors are associated with the development of oral and oropharyngeal cancers, such as malnutrition, sanitary problems and human papillomavirus (HPV). The HPV-16 genotype was identified as a causative agent in many patients with oropharyngeal cancer, which also causes cervical cancer [18–20]. However, oncogenicity associated with malnutrition, sanitary problems and human papillomavirus (HPV) were not as prominent as drinking and smoking in terms of causative agents. We speculate that such difference in risk factors may explain why hypopharyngeal and laryngeal cancer were risk factors of SPCs.

It is generally considered that cancer incidence continuously increases with increasing age, which is compatible with the age-specific incidence risk in Japan, according to the cancer registry and statistics [3]. Furthermore, in a previous study, the cumulative risk of metachronous SPC was correlated with age at diagnosis of the index cancer [5]. Therefore, a higher incidence of metachronous esophageal cancer in young patients at diagnosis of index HNCs was an extremely exceptional and novel finding. One large cohort study demonstrated that the standardized incidence ratio (SIR) of second primary esophageal cancer was highest in young patients (< 56 years) [21]. SIR is a relative incidence of cancer with reference to the general population. Based on the results of this study as well as our study, young patients with HNCs may possess a higher risk of esophageal cancer than the general population and old patients with HNCs.

Alcohol [12, 16, 22–25], cigarette smoke [12, 16, 24], and alcohol metabolizing enzyme deficiencies such as aldehyde dehydrogenase-2 (ALDH2) [12, 26] are the main risk factors for HNC and esophageal cancer. Enzyme deficiencies as well as duration and density of exposure to alcohol and cigarette smoke may determine the risk of these cancers [12, 24]. We assume that young patients with HNCs are exposed to alcohol and cigarette smoke at high carcinogenic levels for a short duration. As a result of this type of exposure to the risk factors, HNC may develop in the younger population. Alcohol and cigarette smoke are the main risk factors for esophageal cancer as well as HNC. High levels of exposure to the risk factors in young patients with HNCs may explain why the cumulative incidence of metachronous esophageal cancer following HNCs was higher in young-onset patients. On the other hand, other cancers such as lung cancer, gastric cancer and colon cancer have different risk factors from HNC. Accordingly, the incidence of other cancers increased with increasing age, because rapid exposure to alcohol and cigarette smoke does not significantly affect the incidence of other cancers.

There are several limitations in our study. First, this was a retrospective study conducted at a single center. However, using cancer registry data, we could accumulate reliable data on the incidence of cancer. Second, the cancer registry is specialized, and information on drinking and smoking habits were not recorded. Therefore, we could not analyze the data if age was an independent risk in the multivariate analysis including drinking and smoking habits. The results of our study are important from an epidemiological point of view. SPCs frequently develop in HNC patients with an annual incidence of 3–4% [6–8] and affect the survival of HNC patients [6, 7]. Early detection of SPCs using strict surveillance is required for the improvement of survival. Based on our results, we can stratify the risk of cancer: young patients (< 65 years) at a high risk for esophageal cancer and old patients (≥65 years) at a high risk of cancers other than esophageal and lung cancer. Stratifying the risk by age is simple, easy, and objective when compared to other risk factors. Furthermore, our results can be used for patient education, i.e., to enlighten young patients that they are at a high risk of developing esophageal cancer.

## Conclusions

We have identified some predictors of SPCs after HNCs. Stratifying the risk by age and other predictors may enable effective surveillance of patients with HNCs.

## Abbreviations

ALDH2: Aldehyde dehydrogenase-2
HNC: Head and neck cancer
HPV: Human papillomavirus
SIR: Standardized incidence ratio
SPC: Second primary cancer
